# Successful institutional care for behavioral and psychological symptoms of dementia in a repopulated area after the 2011 Fukushima disaster: A case report

**DOI:** 10.1002/ccr3.1867

**Published:** 2018-10-17

**Authors:** Yoshitaka Nishikawa, Hidehito Niimura, Akihiko Ozaki, Yuko Kimura, Tomohiro Morita, Toyoaki Sawano, Hiroaki Saito, Masaharu Tsubokura

**Affiliations:** ^1^ Kawauchi Intensive Care Homes for the Elderly Futaba‐gun Japan; ^2^ Kawauchi Village National Health Insurance Clinic Futaba‐gun Japan; ^3^ Department of Internal Medicine Hirata Central Hospital Ishikawa‐gun Japan; ^4^ Department of Health Informatics, School of Public Health Kyoto University Kyoto Japan; ^5^ Department of Neuropsychiatry Keio University School of Medicine Tokyo Japan; ^6^ Department of Breast Surgery Jyoban Hospital of Tokiwa Foundation Iwaki Japan; ^7^ Department of Epidemiology and Biostatistics Teikyo University Graduate School of Public Health Tokyo Japan; ^8^ Department of Internal Medicine Soma Central Hospital Soma Japan; ^9^ Department of Surgery Minamisoma Municipal General Hospital Minamisoma Japan; ^10^ Department of Public Health Fukushima Medical University School of Medicine Fukushima Japan; ^11^ Department of Gastroenterology Sendai Kousei Hospital Sendai Japan

**Keywords:** dementia, Fukushima nuclear accident, long‐term care, nursing homes, social capital

## Abstract

Caregiving in a long‐term facility played a key role in improvements of this patient's behavioral and psychological symptoms of dementia, which also led to a reduced caregiver burden on her family members. Considering the global population aging trend, the lesson from this case may apply to other settings beyond disasters.

## 1 INTRODUCTION

In March 2011, Fukushima, Japan, experienced a nuclear disaster. An 81‐year‐old woman was diagnosed as behavioral and psychological symptoms of dementia (BPSD) during a long‐term evacuation in June 2012; thereafter, her cognitive ability further worsened further. Yet, admission to a long‐term care facility in her home area alleviated her BPSD.

Behavioral and psychological symptoms of dementia (BPSD) are non‐cognitive symptoms and behaviors, such as hallucinations, agitation, and aggression, and are common among dementia patients.^1^ The prevalence of BPSD is an impending issue because of the increasing number of dementia patients worldwide.^2^, ^3^ There were about 47 million people with dementia worldwide in 2015. More than 50% of dementia patients develop BPSD in their lifetime, cumulatively imposing a marked burden on an aging society.^2^, ^4^ BPSD impair the dementia patients’ daily lives^2^ and can impose a significant caregiver burden.^5^ It is therefore important for BPSD patients to receive formal long‐term care. In limited‐resource areas without abundant informal family‐caregiving resources, there is a particular need for formal caregiving for those with BPSD.^1^ However, there is little information on the provision of care for patients with BPSD in limited‐resource areas.

**Figure 1:**
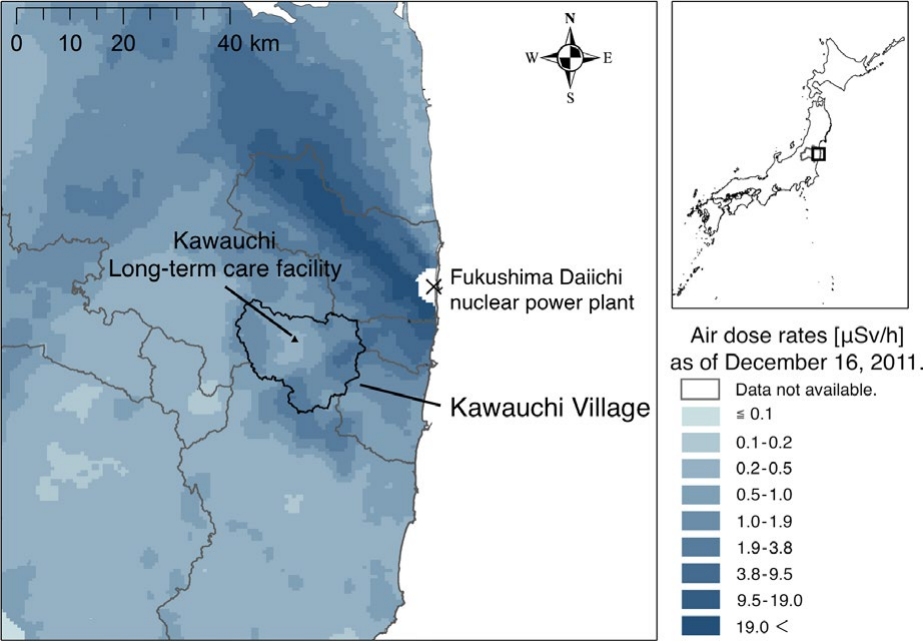
Location of Kawauchi Village, Fukushima, Japan. Kawauchi Village, Fukushima, is located in a mountainous area 10‐30 km southwest of the Fukushima Daiichi nuclear power plant. The Japanese government issued an evacuation order to twelve municipalities, including Kawauchi Village, immediately after the Fukushima nuclear disaster; the local government declared that it was safe to return to the village in January 2012 based on low regional radiation levels. In April 2012, former residents of Kawauchi Village started to repopulate their village. Habitable areas gradually enlarged and all areas of Kawauchi Village were open for repopulation in June 2016. Among the total of 2746 residents before the disaster, 1820 had returned to the village by July 1, 2016. Elderly people accounted for 37.9% of the population in 2015.^26^ The long‐term care facility in Kawauchi Village is the sole inhabitable institution in the village

In March 2011, an earthquake and tsunami struck northeastern Japan, triggering the Fukushima Daiichi Nuclear Power Plant (FDNPP) accident, which forced the evacuation of residents living in surrounding areas.^6^ Kawauchi Village, Fukushima, lies in a mountainous area 10‐30 km southwest of the FDNPP (Figure 1). Even before the disaster, this area had a clinic but no hospital^7^ or facilities providing intensive long‐term care. Because radiation exposure in Kawauchi Village was within the acceptable level for living, the former residents started to repopulate from April 2012. The resident's limited access to health care after the disaster was previously described.^8^ The average age of the local population remaining after this disaster was older because many younger residents did not return out of fear of radiation exposure. This major disaster also created social and environmental challenges. As the mass evacuation changed the residents’ living and social environments, it also affected those with chronic diseases, such as diabetes, dyslipidemia, and impaired motor function.^9^, ^10^, ^11^, ^12^ Along with several chronic diseases, this disaster also exacerbated symptoms in those with dementia.^13^, ^14^ It has been reported that changes to circumstances, such as evacuation, could affect patients’ mental and cognitive conditions.^15^ Both cognitive functions and BPSD deteriorated among patients who experienced this disaster.^13^ A previous study has also shown an association between housing damage and cognitive decline, because of new onset of depression and disruption of social contacts.^16^ In response to the increasing demand for care for the aging population in this impacted village, a long‐term care facility was established in November 2015.

The case of Kawauchi Village involves rapid changes in the age distribution of the local population combined with limited age‐care resources; it is thus representative of other areas affected by nuclear disaster. Here, we report on a patient who developed dementia after the Fukushima nuclear disaster and who received long‐term care in a new facility established in the repopulated area. This case emphasizes the importance of providing formal care for patients with BPSD, and their family members, in limited‐resource areas.

## 2 CASE REPORT

An 81‐year‐old woman with dementia was admitted to the long‐term care facility in Kawauchi Village after the 2011 Fukushima disaster. Before the disaster, she had lived in Kawauchi Village with her husband and their son's family. She had experienced a stroke about twenty years ago without neurological sequelae and had no other remarkable past medical history. On March 11, 2011, the Great East Japan Earthquake struck the area, triggering the FDNPP accident. The patient and her husband evacuated to a shelter apart from other family members because of the mandatory evacuation order. After moving to temporary housing in Koriyama, she lived alone with her husband, while some relatives lived in temporary housing nearby.

In June 2012, she presented with memory loss. Physical examination revealed no neurological findings. Her symptoms were stable and were followed up in an outpatient clinic. A cognitive function test had not been performed before the disaster, but the patient and her family did not notice dementia symptoms at that time. Symptoms including agitation, irritability, aggression, and personality change emerged in October 2012, particularly during attendance at daycare activities. At that time, the patient's score on the Mini‐Mental State Examination (MMSE) was 14 out of 30, indicating dementia. At first, she was suspected of having Pick disease because of the personality change. Brain computed tomography revealed hippocampal atrophy without frontotemporal lobar degeneration. Brain magnetic resonance imaging (MRI) revealed a previous putamen lacunar infarction, which could not completely rule out vascular dementia. Brain MRI also showed hippocampal‐dominant atrophy that was 14.6 times as atrophic as other parts of the brain. The neurological changes accompanied by aggressive behavior were consistent with severe BPSD. Together with clinical symptoms and the results of MRI, the diagnosis was confirmed as Alzheimer's disease.

The patient was treated with memantine 10 mg/d, but the BPSD, such as undressing, hiding or stealing objects, agitation, and aggression, continued to worsen. Although the family members made their best effort to provide informal care to the patient, as the patient and her husband lived apart from the family members in the temporary housing, the family's support alone could not cover the entire care of the patient. In February 2015, on order of the designated psychiatrist, she was mandatorily hospitalized for medical care and protection. At the time of admission, the patient's cognitive function could not be measured. Although its use is controversial in dementia patients, percutaneous endoscopic gastrostomy^17^ was performed because she suddenly began to refuse oral intake in September 2015; this decision was based on the family's values and preferences. The patient's BPSD, including agitation and aggression, were slightly alleviated and she was transferred to the nonpsychiatric ward. In January 2016, she was moved to the long‐term care facility in Kawauchi Village with her husband.

At the time of the patient's admission to the long‐term care facility, she was immobilized and needed constant care. She could not understand any explanations or instructions and her cognitive function could not be measured. Although she could not understand explanations, she could swallow and exhibited only mild dysphagia in a modified water‐swallowing function test (MWST) and scored 3b (swallowed successfully but with wet hoarseness).^18^ She was started on oral intake of semisolid foods in addition to gastronomy feeding.

She was admitted to the facility with her husband, and they spent the daytimes together while she received care. Some caregivers in the facility had been raised in Kawauchi Village and had known the patient long before her admission. These caregivers conversed with the patient about life in Kawauchi and their mutual acquaintances. Her son and relatives living nearby continued to visit the facility several times a week and took the patient in a wheelchair for walks around the facility grounds. Caregivers respected the patient's motivation to continue her daily activities such as dressing, bathing, and eating. Her swallowing function improved to an MWST score of 4 (swallowed successfully with no choking or wet hoarseness) in June 2016, when she ate all meals orally without a gastronomy feeding tube.

The patient's BPSD improved sufficiently that the gastronomy feeding was successfully removed in March 2017. She still exhibited cognitive impairment (MMSE: 13) but without severe BPSD by December 2017. She ate meals orally, used a wheelchair independently, and lived with her husband in the long‐term care facility in the repopulated village.

## 3 DISCUSSION

This patient's BPSD improved after her admission to a long‐term care facility in a repopulated village following the 2011 Fukushima disaster. Despite development of dementia and further deterioration after the 2011 Fukushima disaster, she was able to receive enhanced support in the facility from caregivers already known to the patient and the patient's relatives who lived nearby. The caregiver burden experienced by family members was thus offset by the facility.

This case report showed that medication along with long‐term care in a facility located in the patient's home area helped to manage the patient's BPSD and improve her eating function. First, she received care provided with respect and dignity by caregivers, which might have helped alleviate the BPSD, as previously noted in Japanese research.^19^ Some of the institutional caregivers were originally from the patient's village and conversed with her about topics related to the village and their mutual acquaintances, which seemed to provide mental support. In addition, family members visited the facility frequently because it was located in the repopulated village. Furthermore, the patient spent daytimes with her husband in the facility, as a previous report suggested that group migration mitigated the loss of cognitive function even after a disaster.^20^ Building social capital along with antipsychopathic treatment has benefits in terms of improving the symptoms of BPSD.^21^ Examples of heightened social capital that may contribute to the alleviation of BPSD symptoms include conversations with caregivers who know the patient well, admission with a family member, frequent visits by relatives who live nearby, and familiar landscapes. In this case, the long‐term care facility played an important role in providing care and aggregating social capital.

This case also suggested that institutional caregiving helped to complement the informal caregiving by family members in the repopulated village after the 2011 Fukushima disaster. Caregiver burden refers to the physical and mental stress of caregivers caused by informal caregiving, which is reportedly associated with health issues such as sleep deprivation, depression, and suicide.^22^ After the 2011 Fukushima disaster, the affected areas faced an increasing need for caregiving because of depopulation and an increase in the aging population.^7^, ^23^ However, because of these demographic changes, it may have been difficult for the patients’ family to provide all of the care needed.^24^, ^25^ In Kawauchi Village, the percentage of residents aged over 65 years has increased from 34.0% before the disaster in 2011 to 37.9% in 2015; and this percentage was higher than Japan's national average of 26.7% in 2015.^26^ Similar to the demand experienced by Kawauchi Village, demand for long‐term care is starting to exceed the supply because of increasing life expectancy and declining fertility rates, particularly in limited‐resource areas. In such areas, there is a need to alleviate caregiver burden experienced by families. Institutional caregiving is a potential solution for decreasing informal care burden on family caregivers in an aging society.

In conclusion, we report on a patient developed dementia and BPSD after the Fukushima nuclear disaster and experienced an alleviation of her BPSD after admission to a long‐term care facility with her husband. Caregiving in the facility also played an important role in reducing caregiver burden on her family members living in the area after the disaster.

## ETHICS STATEMENT

For this type of study, ethics approval by an institutional review board is not required. Written informed consent was obtained from the patient.

## CONFLICT OF INTEREST

None declared.

## AUTHOR'S CONTRIBUTION

YN: drafted the article. HN, AO, YK, TM, TS, HS, RK, and MT: performed the critical revision of the article for important intellectual content. All the authors involved in the interpretation of the case, and conception and design, and final approval of the article.

## References

[1] Cerejeira J , Lagarto L , Mukaetova‐Ladinska EB . Behavioral and psychological symptoms of dementia. Front Neurol. 2012;3:1‐21. 22586419 10.3389/fneur.2012.00073 PMC3345875

[2] Livingston G , Sommerlad A , Orgeta V , et al. Dementia prevention, intervention, and care. Lancet. 2017;390:2673‐2734. 28735855 10.1016/S0140-6736(17)31363-6

[3] Robinson L , Tang E , Taylor J‐P . Dementia: timely diagnosis and early intervention. BMJ. 2015;350:h3029. 26079686 10.1136/bmj.h3029 PMC4468575

[4] Neil W , Bowie P . Carer burden in dementia—assessing the impact of behavioural and psychological symptoms via self‐report questionnaire. Int J Geriatr Psychiatry. 2008;23(1):60‐64. 17603824 10.1002/gps.1839

[5] Rosdinom R , Zarina M , Zanariah MS , Marhani M , Suzaily W . Behavioural and psychological symptoms of dementia, cognitive impairment and caregiver burden in patients with dementia. Prev Med (Baltim). 2013;57:S67‐S69. 10.1016/j.ypmed.2012.12.025 23313789

[6] Ochi S , Tsubokura M , Kato S , et al. Hospital staff shortage after the 2011 triple disaster in Fukushima, Japan‐an earthquake, tsunamis, and nuclear power plant accident: a case of the Soso district. PLoS One. 2016;11(10):e0164952. 27788170 10.1371/journal.pone.0164952 PMC5082811

[7] Nishikawa Y , Tsubokura M , Yamazaki S . Healthcare delivery to a repopulated village after the Fukushima nuclear disaster: a case of Kawauchi village, Fukushima, Japan. Japan Med Assoc J. 2016;59(4):159‐161. PMC5476993 28638753

[8] Nishikawa Y , Ozawa Y , Tsubokura M , et al. Long‐term vulnerability of access to hemodialysis facilities in repopulated areas after the Fukushima Nuclear Disaster: a case report. Oxford Med Case Rep. 2018;7:228‐230. 10.1093/omcr/omy040 PMC6054208 30046451

[9] Nomura S , Blangiardo M , Tsubokura M , Ozaki A , Morita T , Hodgson S . Postnuclear disaster evacuation and chronic health in adults in Fukushima, Japan: a long‐term retrospective analysis. BMJ Open. 2016;6(2):1‐12. 10.1136/bmjopen-2015-010080 PMC4746456 26846896

[10] Tsubokura M , Takita M , Matsumura T , et al. Changes in metabolic profiles after the Great East Japan Earthquake: a retrospective observational study. BMC Public Health. 2013;13:267. 23521922 10.1186/1471-2458-13-267 PMC3614525

[11] Nishikawa Y , Fukuda Y , Tsubokura M , Kato S , Nomura S , Saito Y . Managing type 2 diabetes mellitus through periodical hospital visits in the aftermath of the great east Japan earthquake disaster: a retrospective case series. PLoS One. 2015;10(5):e0125632. 25946187 10.1371/journal.pone.0125632 PMC4422625

[12] Ishii T , Ochi S , Tsubokura M , et al. Physical performance deterioration of temporary housing residents after the Great East Japan Earthquake. Prev Med Rep. 2015;2:916‐919. 26844168 10.1016/j.pmedr.2015.10.009 PMC4721442

[13] Furukawa K , Ootsuki M , Kodama M , Arai H . Exacerbation of dementia after the earthquake and tsunami in Japan. J Neurol. 2012;259(6):1243‐1243. 22127617 10.1007/s00415-011-6329-x

[14] Furukawa K , Ootsuki M , Nitta A , Okinaga S , Kodama M , Arai H . Aggravation of Alzheimer’s disease symptoms after the earthquake in Japan: A comparative analysis of subcategories. Geriatr Gerontol Int. 2013;13(4):1081‐1082. 24131763 10.1111/ggi.12085

[15] Cloyd E , Dyer CB . Catastrophic events and older adults. Crit Care Nurs Clin North Am. 2010;22(4):501‐513. 21095558 10.1016/j.ccell.2010.10.003

[16] Hikichi H , Aida J , Kondo K , et al. Increased risk of dementia in the aftermath of the 2011 Great East Japan Earthquake and Tsunami. Proc Natl Acad Sci U S A. 2016;113(45):E6911‐E6918. 27791093 10.1073/pnas.1607793113 PMC5111665

[17] Löser C , Aschl G , Hébuterne X , et al. Consensus Statement; ESPEN guidelines on Artificial enteral nutrition ‐ percutaneous endoscopic gastrostomy (PEG). Clin Nutr. 2005;24(5):848‐861. 16261664 10.1016/j.clnu.2005.06.013

[18] Horiguchi S , Suzuki Y . Screening tests in evaluating swallowing function. Japan Med Assoc J. 2011;54(1):31‐34.

[19] Makiya H . [Epidemiological research on psychiatric diseases among the elderly in Okinawa Prefecture] Okinawa no ichi nouson ni okeru roujin no seishinshikkan ni kansuru ekigaku kenkyu (in Japanese). Keio Igaku (Journal Keio Med Soc). 1978;55(6):503‐512.

[20] Hikichi H , Tsuboya T , Aida J , et al. Social capital prevents cognitive decline in the aftermath of a natural disaster: a quasi‐experiment from the 2011 great east Japan earthquake and tsunami. Sci Adv. 2017;1:e105‐e113. 10.1016/S2542-5196(17)30041-4 PMC5801543 29430569

[21] Hersch EC , Falzgraf S . Management of the behavioral and psychological symptoms of dementia. Clin Interv Aging. 2007;2(4):611‐621. 18225462 10.2147/cia.s1698 PMC2686333

[22] Adelman RD , Tmanova LL , Delgado D , Dion S , Lachs MS . Caregiver burden. JAMA. 2014;311(10):1052. 24618967 10.1001/jama.2014.304

[23] Ozaki A , Tsubokura M , Leppold C , et al. The importance of family caregiving to achieving palliative care at home. Medicine (Baltimore). 2017;96(46):e8721. 29145313 10.1097/MD.0000000000008721 PMC5704858

[24] Morita T , Leppold C , Tsubokura M , Nemoto T , Kanazawa Y . The increase in long‐term care public expenditure following the 2011 Fukushima nuclear disaster. J Epidemiol Community Health. 2016;70(7):738. 27288528 10.1136/jech-2015-206983

[25] Ozaki A , Shimada Y , Yamamoto K , et al. Death of the sole doctor at Takano Hospital 6 years after the Fukushima nuclear crisis—who is responsible for health care delivery in the Fukushima disaster zone? QJM An Int J Med. 2018;111(2):79‐81. 10.1093/qjmed/hcx050 28339717

[26] Kawauchi Village . About Kawauchi Village: Population (in Japanese). https://www.kawauchimura.jp/page/page000073.html. Accessed September 12, 2018.

